# Lessons from an initiative to address gender bias

**DOI:** 10.7554/eLife.75818

**Published:** 2021-12-15

**Authors:** Kate E Hoy, Bernadette M Fitzgibbon, Anna-Katharine Brem

**Affiliations:** 1 Epworth Centre for Innovation in Mental Health, Epworth Healthcare and Monash University Department of Psychiatry Camberwell Australia; 2 Department of Epidemiology and Preventive Medicine, School of Public Health and Preventive Medicine, Monash University Melbourne Australia; 3 University Hospital of Old Age Psychiatry Bern Switzerland; 4 Department of Neuropsychology, Lucerne Psychiatry Lucerne Switzerland; 5 Berenson-Allen Center for Noninvasive Brain Stimulation, Division of Interventional Cognitive Neurology and Department of Neurology, Beth Israel Deaconess Medical Center, Harvard Medical School Boston United States

**Keywords:** gender bias, research culture, equity, diversity, inclusion, brain stimulation, conferences, point of view, None

## Abstract

How a letter addressing the lack of women invited to speak at a conference in brain stimulation encouraged researchers to take action.

The 1^st^ International Brain Stimulation Conference, held in Singapore in March 2015, was eagerly anticipated. Organized by the journal *Brain Stimulation* it was to be the largest international conference in the field. For many attendees, however, it was more notable for the fact that just two of the 39 invited speakers were female (Hoy, 2017; Brain Stimulation, 2015). Giving talks at conferences is important for building a career in research, not inviting women to speak makes it harder for them to progress, adding to the other forms of gender bias that are already present in the research system.

Following the Singapore meeting, the lack of gender balance was brought to the attention of the conference organizers. However, for the next meeting in the series, to be held in Barcelona in March 2017, the problem was even worse – all 10 invited speakers were male (Brain Stimulation, 2017). Consequently, the website biaswatchneuro.com rated the conference a zero – signifying that the percentage of female speakers was more than two standard deviations below the estimated percentage of women in the field (which was 34 % at the time; BiasWatchNeuro.com, 2016).

The problems with gender balance at the 2015 conference, and the announcement of the all-male invited speaker line up for the 2017 conference, prompted one of the present authors (KEH) to submit a letter to the editors of *Brain Stimulation* (Hoy, 2017). The purpose of the letter, which was published under the title "Gender imbalance at brain stimulation conferences: We have a problem and it is everyone’s problem", was three-fold: (i) to raise awareness of the issue and to start a conversation about diversity of representation in brain stimulation; (ii) to highlight the tangible and intangible impacts of lack of diversity, not only on those excluded, but also on the progress of the field as a whole; (iii) to propose constructive and practical solutions. The solutions proposed were simple: first, to compile and implement visible speaker policies, examples of which are widely and freely available (such as those used by the Society of Crystallographers in Australia and New Zealand and by Nature Conferences); second, to build a database website called womeninbrainstim.com to help conference organizers identify female researchers who could be invited to speak at conferences (see Box 1; Figure 1).

The letter succeeded in raising awareness and sparked a robust conversation that included a number of published responses in *Brain Stimulation* (George and Sackeim, 2017; Hinder et al., 2017; Antal, 2017) and an editorial in another journal (Brem et al., 2017). This episode also encouraged other researchers in the field to take action, and even resulted in some of the delegates at the second conference adding protest signs to poster presentations (Figure 1).

Box 1. **The Women in BrainStim database**

Womeninbrainstim.com was launched in November 2016 with the aim to help conference organizers, journal editors, universities and other institutions identify female researchers working in brain stimulation. Each profile contains information on career stage, area of research, techniques used, as well as top five papers and awards; making it easy for users to find scientists with specific skills or experiences. The design, implementation, launch and promotion of the site were strategically planned in order to achieve significant impact within the shortest time frame possible (see ‘Top tips for impact’ below). As of November 2021, there are 326 scientists from 31 countries registered on the site (Figure 2): 31.6 % indicated they are a ‘Principal Investigator’, 26.4 % identified as ‘Post-Doctoral’ and 41.4 % as ‘Other’ (i.e., student or in research assistant type roles). Less than 1 % did not indicate career stage. While there are a number of existing databases of female scientists, such as Request a Women Scientist and Gage.500 Women Scientists, with similar aims to that of the Women in BrainStim site, a field specific resource allows for more direct engagement with the field and hopefully greater participation from all stakeholders. At the end of 2019, a survey of users was conducted to explore how the site could be further developed to advance equity, diversity and inclusion more broadly in the field. Based on the rich user feedback provided, there are plans to improve and extend the functionality of the site and the community that has developed for the benefit of all users.

**Speaker database sites: Top tips for impact**

- Seek professional advice/assistance with the design and development. Usability testing, or informal user feedback at minimum, should be conducted early in the process.
- A targeted ’soft’ launch is recommended to gain momentum and increase credibility. Contact leaders in the field before launch and ask them to register.
- Implement a promotion strategy at the time of launch. This can include asking colleagues to share the database via their networks, advertising widely on social media (social media share buttons can be added to the site to allow easy ‘one click’ promotion), and having a conference slide that users can include in their presentations to promote the database.
- To keep up momentum after launch, call on people to join and utilize the site regularly on social media channels; this can be particularly useful when attached to relevant campaigns/events and using their hashtag (e.g., International Women’s Day, or the lead up to the field’s major conference).
- It is important to monitor, evaluate and (where needed) improve the site to ensure that it stays relevant and useful. Inclusion of a feedback form can assist with this.

**Figure 1.**
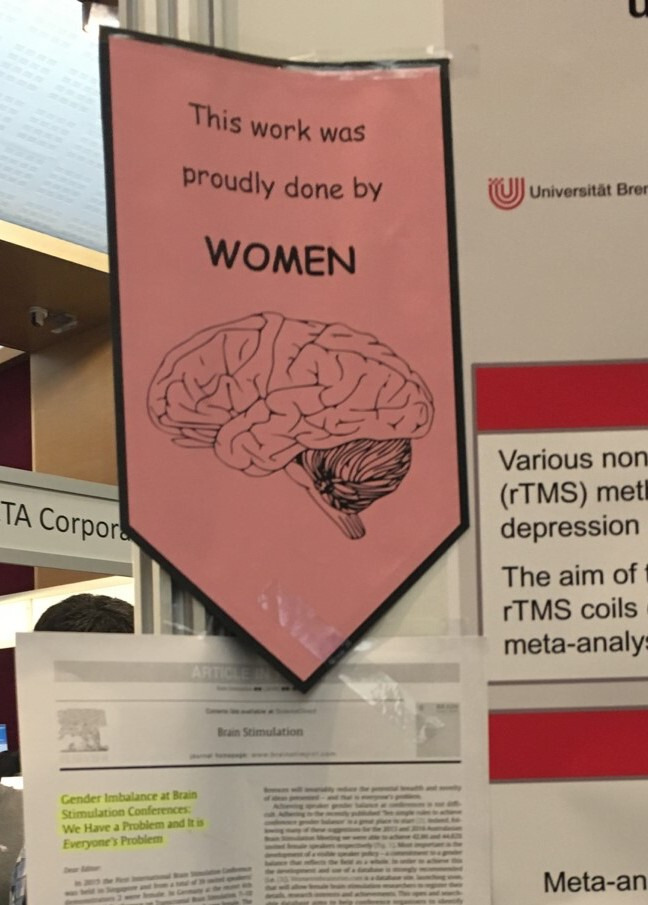
**This work was proudly done by WOMEN**. A poster at the 2^nd^ International Brain Stimulation Conference in Barcelona in 2017.

Image credit: Photo by Dr Melanie Emonson, sign created by Dr Karina De Santis and Ms Brigitte Ernst.

**Figure 2.**
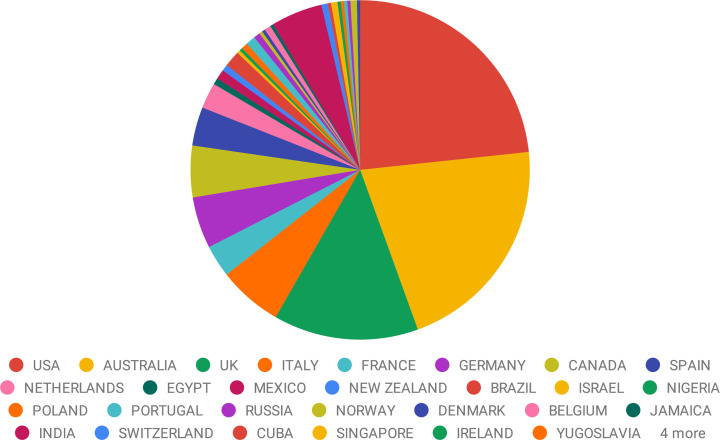
Geographic location of researchers in the database. Geographic distribution of the 326 registered scientists on the “Women in BrainStim” database as of November 2021.

It is worth stressing that achieving a gender balanced speaker line up, while often elusive, is not actually difficult. The steps that need to be taken are well known, simple and have been published in an open-access journal by Professor Jenny Martin: her article, "Ten simple rules to achieve conference speaker gender balance" is a literal step-by-step guide (Martin, 2014). The problem is not that conference organizers do not know what to do: rather, they often seem to be unwilling to take action and do what is required. The growing number of gender balanced brain stimulation conferences (see Box 2 for examples) clearly illustrates that it is possible to ensure a representative speaker line up when conference organizers are genuinely committed to speaker gender diversity.

Box 2. **Gender balance at conferences in brain stimulation.**

The number of brain stimulation conferences with a balance of male and female speakers is steadily increasing. Indeed, at the first and second Australasian Brain Stimulation Meetings, which were held in 2013 and 2016, 45.6 % and 43.2 % of the speakers were female, and the Australasian Brain Stimulation Society, established in 2018, has consistently maintained representative speaker line ups in all its events.

Prompted by what happened in Barcelona, the organizers of the second European Conference in Brain Stimulation in Psychiatry (ECBSP) in Munich introduced a speaker policy and a gender score, both of which appeared on the conference website. The conference achieved a representative 40 % female speaker participation. The importance of gender balance was also discussed in the opening session of the conference and was the subject of an editorial in a leading journal in the field (Brem et al., 2017). The gender balance was even better at the third conference in the series, with almost 49 % of all presenters being female (Brunelin et al., 2019).

The organizers of the Carolina Neurostimulation conference, first held in May 2018, also responded to the absence of female speakers at the Barcelona meeting, writing on their Facebook page that they wanted to “bring in different perspectives typically not heard” and to ensure that they were not asking the same people who already have a (magnified) voice in the brain stimulation community. This resulted in an almost 50:50 speaker balance (10 female speakers and 11 male speakers), and a line up of speakers who were at different career stages.

These examples are not meant to be exhaustive; rather they are presented as examples to show that it is possible to organize conferences that are diverse in terms of speakers. The gender balance of the International Brain Stimulation Conference has also improved. Four of the 11 named plenaries at the third conference (Brain Stimulation, 2019), and five of the 12 speakers at the fourth conference (held in Charleston in December 2021) were female.

The fight for gender balance at conferences is of course not unique to the field of brain stimulation. A recent analysis of gender balance among invited speakers at conferences in five different fields found that gender balance appeared to be improving in four of the five areas: in neuroscience, for example, the percentage of female speakers at the meetings studied had increased from 24 % in 2011 to 42% in 2019 (Else, 2019). This indicates that, consistent with the brain stimulation examples provided here, it is possible to achieve balance and progress is being made.

Attempts to change the status quo often encounter resistance, so those calling for action should be prepared to respond to such resistance. For example, one response to Hoy’s original letter about gender imbalance argued that "the highest priority was and will continue to be scientific merit" (George and Sackeim, 2017). This argument is often made when efforts are made to address bias in science. However, Hinder et al. were able to refute this claim by showing that, for the most highly cited articles published in *Brain Stimulation* between 2014 and 2016, 44 % of the first authors and 26 % of the senior authors were female (Hinder et al., 2017), thus demonstrating that committing to a more gender balanced conference would not have in any way sacrificed quality.

So how can conference organizers be held to diversity standards? Where there is no attempt to address gender balance, invited speakers can refuse to participate. Similarly, potential attendees can ask conference organizers to share the speaker policy or even choose not to attend non-diverse meetings. However, it would be better if the scientific societies, research institutions, publishers and other bodies that own and run conferences were proactive and ensured that all the relevant committees were gender balanced, that speaker policies were visible, and that speaker line ups represented the diversity of the field.

While gender balance in committees is important, it does not guarantee gender balance among speakers. It is therefore vital to also implement a speaker policy. As an example, a short sentence, such as "Symposia proposals should respect the gender equality policy of the conference (equal gender representation for speakers and for chairpersons)", can be included in the template for symposium submissions. Furthermore, the organizing committees should be dedicated to selecting equal gender representation of contributors for oral and poster presentations.

The current rise in virtual conferences may facilitate the inclusion of women with carer responsibilities as it reduces the overall amount of time dedicated to a conference (e.g., travel time, time spent networking). This economy of time (and the general lack of childcare facilities at conferences) may lead more women to accept an invitation to speak at a conference. However, virtual conferences can still present inclusion challenges dependent on their location (time zone) and concurrent carer responsibilities. It is important to note that irrespective of the conference format – i.e. virtual versus in person – organizers need to be willing to adhere to a speaker policy which will ensure diversity and inclusion.

Finally, and critically, efforts to increase diversity in science cannot be only focused on gender, true diversity requires complete representation and inclusion of people with all lived experiences. We must commit to always having diverse and inclusive editorial boards, conference committees, speaker line ups and award panels to ensure any hard-fought gains made are maintained and embedded. Above all we must all continue to press for progress.
